# Supplementary material for: A Proximity Complementation Assay to Identify Small Molecules That Enhance the Traffic of ABCA4 Misfolding Variants
Source: Int J Mol Sci. 2024 Apr 20;25(8):4521. doi: 10.3390/ijms25084521 (PMC11050442; doi:10.3390/ijms25084521)
Supplement: Supplementary file 1 [file ijms-25-04521-s001.zip › ijms-2918545-supplementary.pdf]

Supplementary Material

A proximity complementation assay to identify small molecules that enhance the traffic of ABCA4 misfolding variants.

Davide Piccolo, Christina Zarouchlioti, James Bellingham, Rosellina Guarascio, Kalliopi Ziaka, Robert S. Molday and Michael E. Cheetham

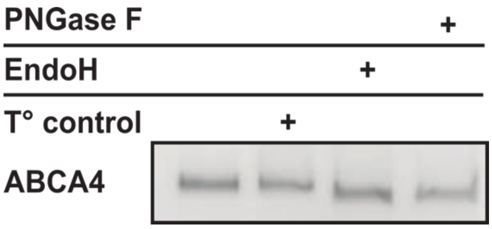

Figure S1. Glycosylation status of ABCA4 in mouse retina. 6 µg of protein lysate from mouse retina were treated with EndoH, PNGase-F, or buffer and temperature only protocol-control (T°). A slight increase in protein mobility was observed following both PNGase-F and EndoH. N= 2 mice retinas.

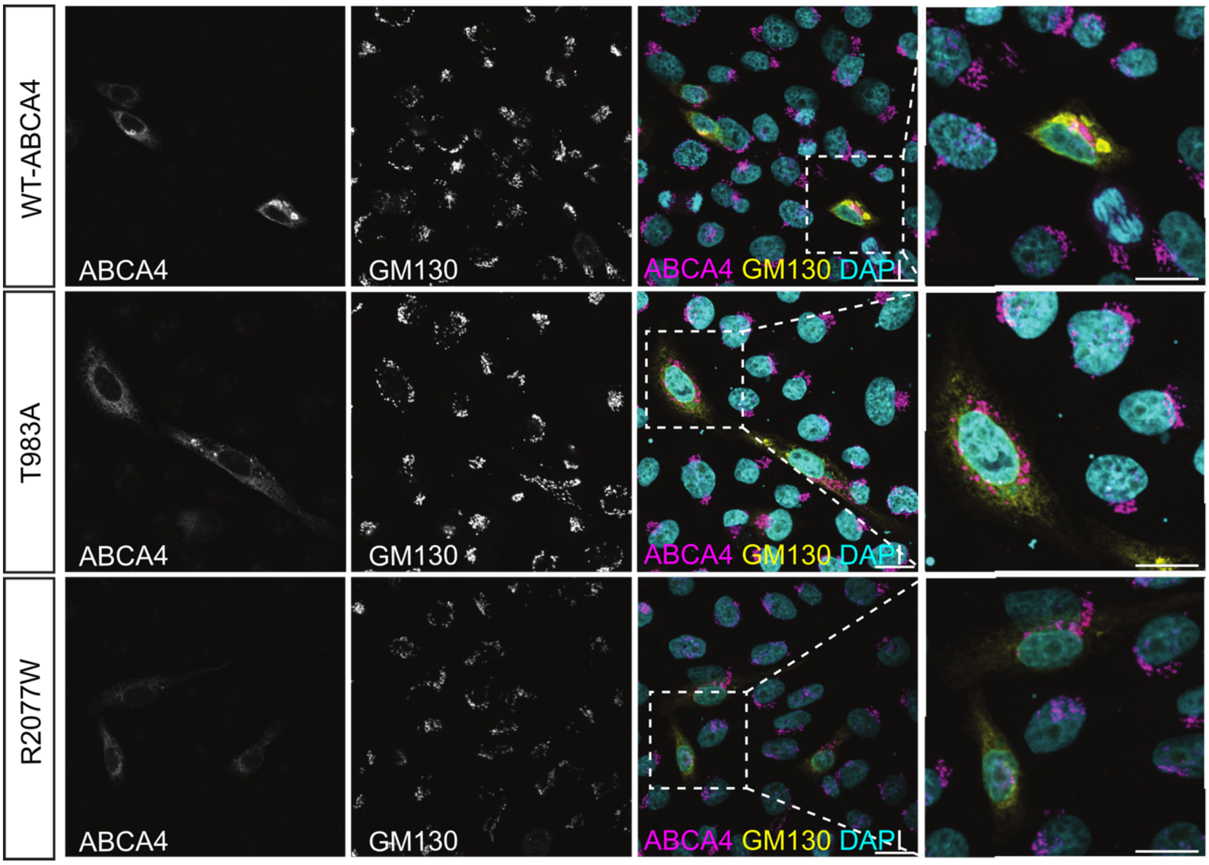

Figure S2. ABCA4 and Golgi localization in cultured cells. CHO cells were transfected with plasmid expressing WT ABCA4 protein and the indicated variants. 48 hours post-transfection cells were fixed in 4% PFA and permeabilised with Triton X-100 and double labelled with the ABCA4-Abbexa (yellow) and GM130 (magenta). ABCA4 was not detected overlapping with the Golgi. Scale bars = 10 µm

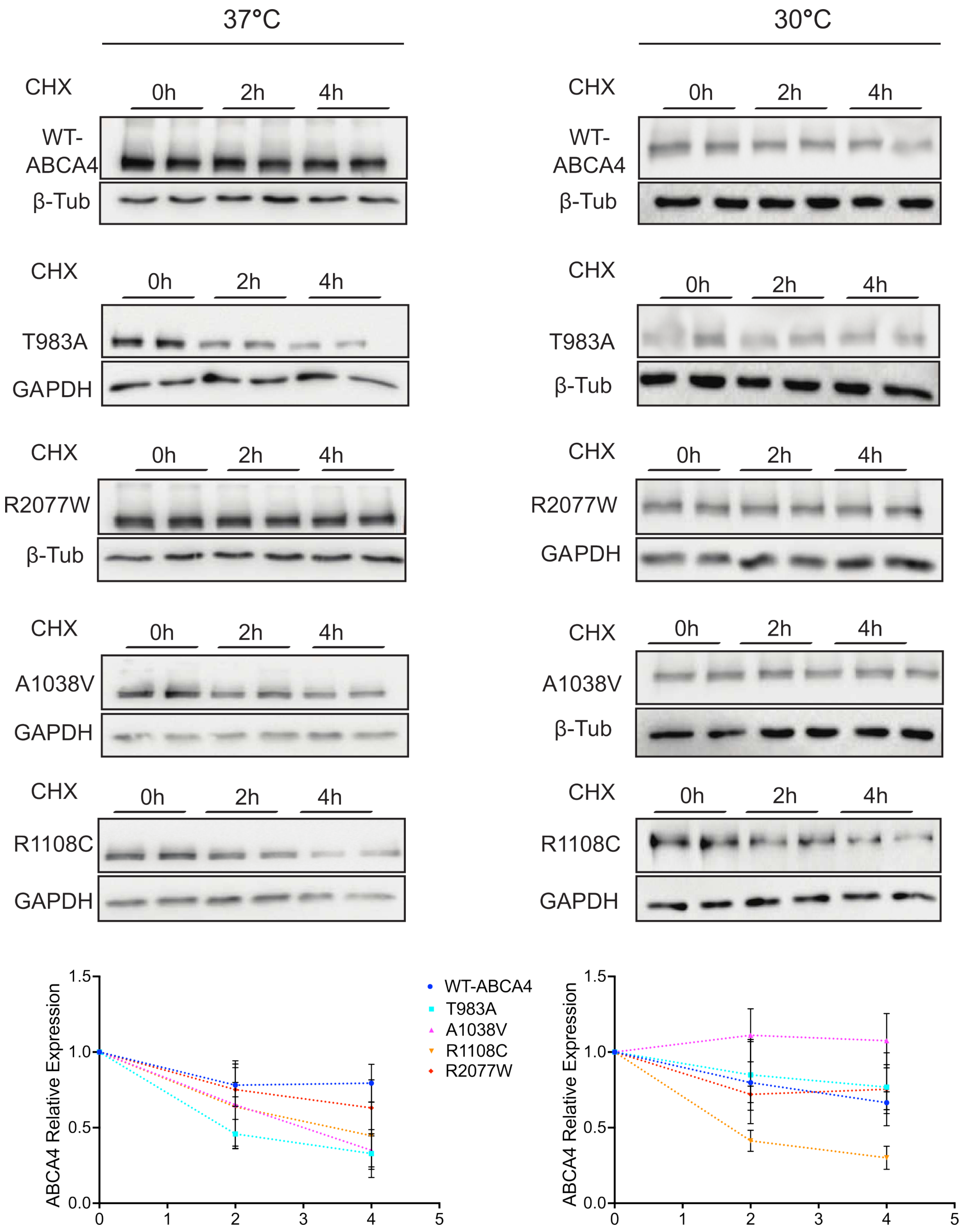

Figure S3. The effect of the temperature on ABCA4 protein degradation. HEK293T cells were transfected with plasmids expressing WT ABCA4 and variants and incubated at 37ºC or 30ºC. 48h post-transfection cells were treated with 50 μM of (CHX) for 0, 2 and 4 h and western blotted (10 μg of protein lysate). Graphs show quantification of protein levels by ImageJ. Data were normalised to GAPDH/β-Tubulin. Mean of fold change + SD. n = 3 independent experiments.

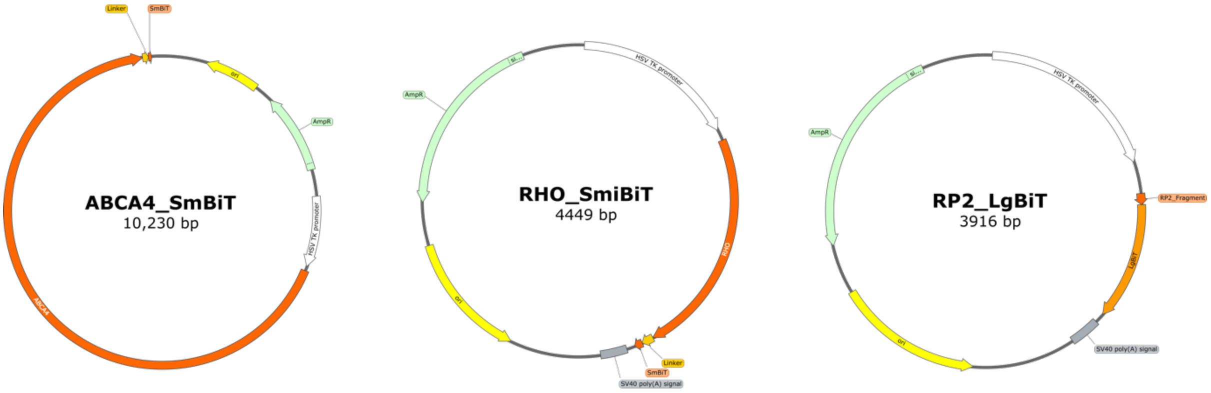

Figure S4. Plasmid maps. The RP2-LBiT, RHO-SmBiT and ABCA4-SmBiT plasmid maps. The P23H and the ABCA4 missense variants are not shown since they all are single nucleotide variants in the coding sequence and the vector backbones are the same.

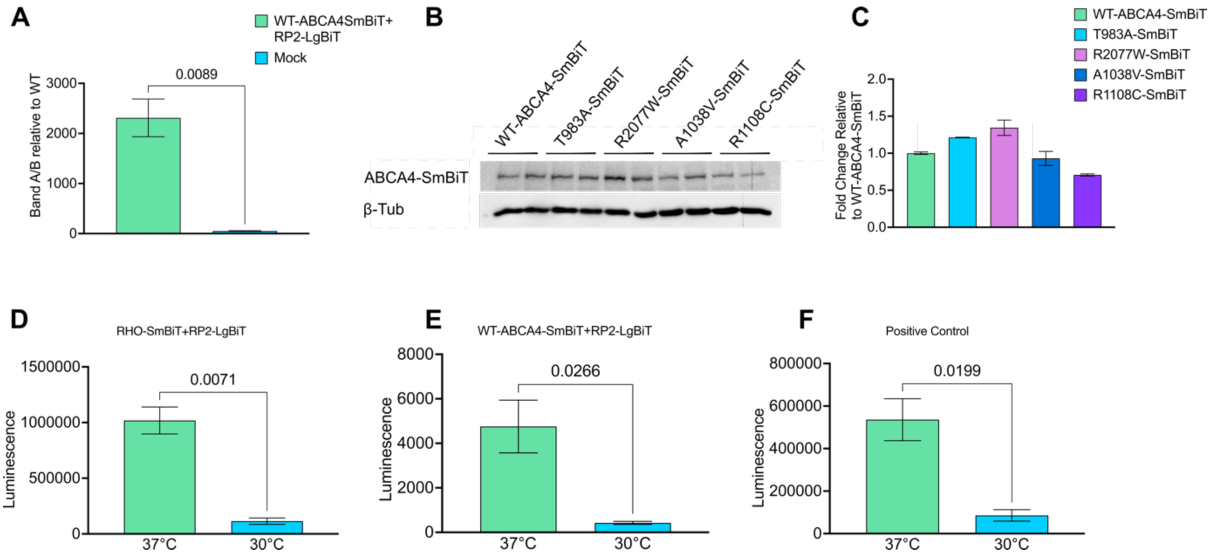

Figure S5. Validation of split NanoBit complementation assay A) 48h post-transfection live HEK293T cells were analysed using a luminometer. WT-ABCA4-SmBiT + RP2-SmBiT shows a stronger luminescence with compared to the non-transfected cells (Mock). Error bars are SD. n = 3. Two-tailed Student's t-test. **B,C**) 48h post-transfection HEK293T cells were collected, and the protein lysate was analysed by western blot and quantification was obtained with ImageJ. No marked decrease in the steady state level of the ABCA4 proteins was detected. n = 2. **D-F**) Luminescence produced at 37ºC was compared to the signal produced at 30ºC using the RHO-SmBiT+RP2-LgBiT and the ABCA4-SmBiT+RP2-LgBiT plasmids in HEK293T. A significant drop in luminescence signal was observed at 30ºC in HEK293T cells in both conditions. **F**) Luminescence signal at 37ºC vs 30 ºC was also studied using the positive control plasmids provided in the NanoLuc Binary Technology system by Promega. A significant drop in luminescence signal was observed at 30ºC. Error bars are SD. Two-tailed Student's t-test.

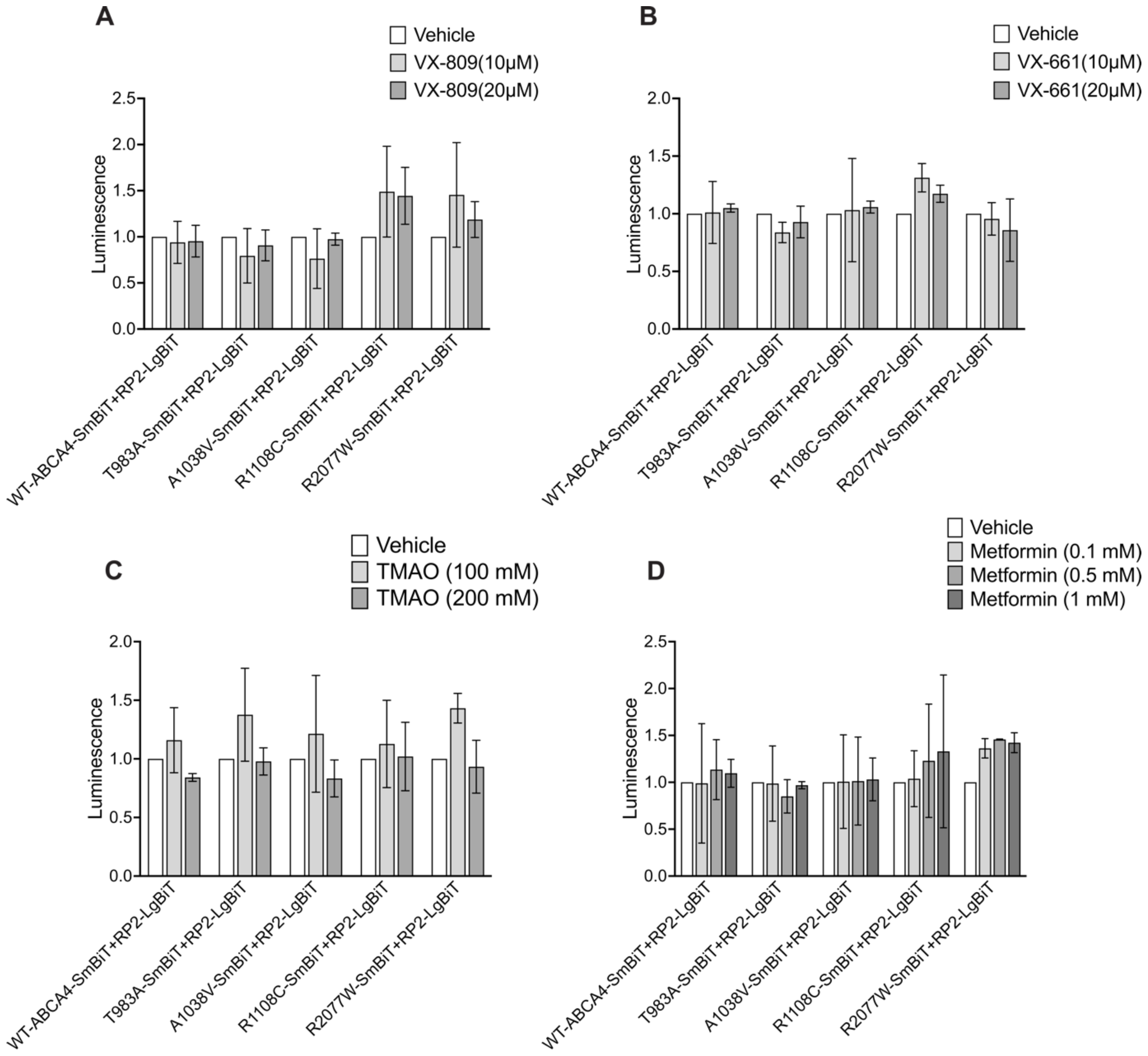

Figure S6. Small compounds are screened using the complementation assay. A and **B**) Luminescence signal in HEK293T was analysed after 48h from transient transfection for the indicated NanoBiT fusion combinations. 24h post transfection cells were treated with 10 μM or 20 μM of VX-809 (**A**) and VX-661 (**B**) for a total of 24h treatment. Mean fold change in luminescence relative to the vehicle + SD. n=2. **C**) 48h post transfection cells were treated with different concentrations of TMAO (100 and 200 mM) for a total of 24h treatment. Mean fold change in luminescence relative to the vehicle + SD. n=3. **D**) Luminescence signal in HEK293T was analysed after 48h from transient transfection for the indicated NanoBiT fusion combinations. 24h post transfection cells were treated with different concentration of metformin (0.1, 0.5 and 1 mM) for a total of 24h treatment. Mean fold change in luminescence relative to the vehicle + SD. n=2

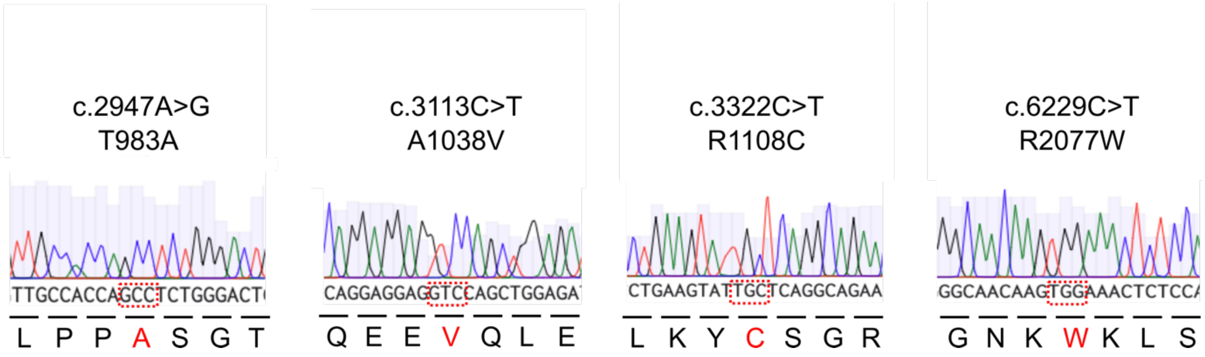

Figure S7. ABCA4 variants sequences. Sanger sequences of ABCA4-SmBiT plasmid variants to confirm the introduction of the specific point mutation in the correct position (red box).

WT-ABCA4-SmBiT plasmid

TTGTGATGCTATTGCTTTATTTGTAACCATTATAAGCTGCAATAAACAAGTTAACAACAAC
AATTGCATTCATTTTATGTTTCAGGTTCAGGGGGAGGTGTGGGAGGTTTTTTAAAGCAA
GTAAAACCTCTACAAATGTGGTAAAATCGATAAGGATCCGTCGACCGATGCCCTTGAGA
GCCTTCAACCCAGTCAGCTCCTTCCGGTGGGCGCGGGGCATGACTATCGTCGCCGCA
CTTATGACTGTCTTCTTTATCATGCAACTCGTAGGACAGGTGCCGGCAGCGCTCTTCCG
CTTCCTCGCTCACTGACTCGCTGCGCTCGGTCGTTCGGCTGCGGCGAGCGGTATCAG
CTCACTCAAAGGCGGTAATACGGTTATCCACAGAATCAGGGGATAACGCAGGAAAGAA
CATGTGAGCAAAAGGCCAGCAAAAGGCCAGGAACCGTAAAAAGGCCGCGTTGCTGGC
GTTTTTCCATAGGCTCCGCCCCCCTGACGAGCATCACAAAAATCGACGCTCAAGTCAGA
GGTGGCGAAACCCGACAGGACTATAAAGATACCAGGCGTTTCCCCCTGGAAGCTCCCT
CGTGCGCTCTCCTGTTCCGACCCTGCCGCTTACCGGATACCTGTCCGCCTTTCTCCCTT
CGGGAAGCGTGGCGCTTTCTCATAGCTCACGCTGTAGGTATCTCAGTTCGGTGTAGGT
CGTTCGCTCCAAGCTGGGCTGTGTGCACGAACCCCCCGTTCAGCCCGACCGCTGCGC
CTTATCCGGTAACTATCGTCTTGAGTCCAACCCGGTAAGACACGACTTATCGCCACTGG
CAGCAGCCACTGGTAACAGGATTAGCAGAGCGAGGTATGTAGGCGGTGCTACAGAGTT
CTTGAAGTGGTGGCCTAACTACGGCTACACTAGAAGAACAGTATTTGGTATCTGCGCTC
TGCTGAAGCCAGTTACCTTCGGAAAAAGAGTTGGTAGCTCTTGATCCGGCAAACAAACC
ACCGCTGGTAGCGGTGGTTTTTTTGTTTGCAAGCAGCAGATTACGCGCAGAAAAAAAG
GATCTCAAGAAGATCCTTTGATCTTTTCTACGGGGTCTGACGCTCAGTGGAACGAAAAC
TCACGTTAAGGGATTTTGGTCATGAGATTATCAAAAAGGATCTTCACCTAGATCCTTTTA
AATTAAAAATGAAGTTTTAAATCAATCTAAAGTATATATGAGTAAACTTGGTCTGACAGC
GGCCGCAAATGCTAAACCACTGCAGTGGTTACCAGTGCTTGATCAGTGAGGCACCGAT
CTCAGCGATCTGCCTATTTCGTTCGTCCATAGTGGCCTGACTCCCCGTCGTGTAGATCA
CTACGATTCGTGAGGGCTTACCATCAGGCCCCAGCGCAGCAATGATGCCGCGAGAGC
CGCGTTCACCGGCCCCCGATTTGTCAGCAATGAACCAGCCAGCAGGGAGGGCCGAGC
GAAGAAGTGGTCCTGCTACTTTGTCCGCCTCCATCCAGTCTATGAGCTGCTGTCGTGAT
GCTAGAGTAAGAAGTTCGCCAGTGAGTAGTTTCCGAAGAGTTGTGGCCATTGCTACTG
GCATCGTGGTATCACGCTCGTCGTTCGGTATGGCTTCGTTCAACTCTGGTTCCCAGCG
GTCAAGCCGGGTCACATGATCACCCATATTATGAAGAAATGCAGTCAGCTCCTTAGGGC
CTCCGATCGTTGTCAGAAGTAAGTTGGCCGCGGTGTTGTCGCTCATGGTAATGGCAGC
ACTACACAATTCTCTTACCGTCATGCCATCCGTAAGATGCTTTTCCGTGACCGGCGAGT
ACTCAACCAAGTCGTTTTGTGAGTAGTGTATACGGCGACCAAGCTGCTCTTGCCCGGC
GTCTATACGGGACAACACCGCGCCACATAGCAGTACTTTGAAAGTGCTCATCATCGGG
AATCGTTCTTCGGGGCGGAAAGACTCAAGGATCTTGCCGCTATTGAGATCCAGTTCGAT
ATAGCCCACTCTTGCACCCAGTTGATCTTCAGCATCTTTTACTTTCACCAGCGTTTCGG
GGTGTGCAAAAACAGGCAAGCAAAATGCCGCAAAGAAGGGAATGAGTGCGACACGAAA
ATGTTGGATGCTCATACTCGTCCTTTTTCAATATTATTGAAGCATTTATCAGGGTTACTA
GTACGTCTCTCAAGGATAAGTAAGTAATATTAAGGTACGGGAGGTATTGGACAGGCCG
CAATAAAATATCTTTATTTTCATTACATCTGTGTGTTGGTTTTTTGTGTGAATCGATAGTA
CTAACATACGCTCTCCATCAAAACAAAACGAAACAAAACAAACTAGCAAAATAGGCTGT
CCCCAGTGCAAGTGCAGGTGCCAGAACATTTCTCTGGCCTAACTGGCCGGTACCTGAG
TCTAAATGAGTCTTCGGACCTCGCGGGGGCCGCTTAAGCGGTGGTTAGGGTTTGTCTG
ACGCGGGGGGAGGGGGAAGGAACGAAACACTCTCATTCGGAGGCGGCTCGGGGTTT
GGTCTTGGTGGCCACGGGCACGCAGAAGAGCGCCGCGATCCTCTTAAGCACCCCCCC
GCCCTCCGTGGAGGCGGGGGTTTGGTCGGCGGGTGGTAACTGGCGGGCCGCTGACT
CGGGCGGGTCGCGCGCCCCAGAGTGTGACCTTTTCGGTCTGCTCGCAGACCCCCGGG
CGGCGCCGCCGCGGCGGCGACGGGCTCGCTGGGTCCTAGGCTCCATGGGGACCGTA

TACGTGGACAGGCTCTGGAGCATCCGCACGACTGCGGTGATATTACCGGAGACCTTCT
GCGGGACGAGCCGGGTCACGCGGCTGACGCGGAGCGTCCGTTGGGCGACAAACACC
AGGACGGGGCACAGGTACACTATCTTGTCACCCGGAGGCGCGAGGGACTGCAGGAGC
TTCAGGGAGTGGCGCAGCTGCTTCATCCCCGTGGCCCGTTGCTCGCGTTTGCTGGCG
GTGTCCCCGGAAGAAATATATTTGCATGTCTTTAGTTCTATGATGACACAAACCCCGCC
CAGCGTCTTGTCATTGGCGAAGTCGAACACGCAGATGCAGTCGGGGCGGCGCGGTCC
CAGGTCCACTTCGCATATTAAGGTGACGCGTGTGGCCTCGAACACCGAGCGACCCTGC
AGCGACCCGCTTAAAAGCTTGGCAATCCGGTACTGTGGTAAAGCCACCAGATCTGGCC
ACCATGGGCTTCGTGAGACAGATACAGCTTTTGCTCTGGAAGAACTGGACCCTGCGGA
AAAGGCAAAAGATTCGCTTTGTGGTGGAACTCGTGTGGCCTTTATCTTTATTTCTGGTCT
TGATCTGGTTAAGGAATGCCAACCCACTCTACAGCCATCATGAATGCCATTTCCCCAAC
AAGGCGATGCCCTCAGCAGGAATGCTGCCGTGGCTCCAGGGGATCTTCTGCAATGTGA
ACAATCCCTGTTTTCAAAGCCCCACCCCAGGAGAATCTCCTGGAATTGTGTCAAACTAT
AACAACTCCATCTTGGCAAGGGTATATCGAGATTTTCAAGAACTCCTCATGAATGCACC
AGAGAGCCAGCACCTTGGCCGTATTTGGACAGAGCTACACATCTTGTCCCAATTCATGG
ACACCCTCCGGACTCACCCGGAGAGAATTGCAGGAAGAGGAATACGAATAAGGGATAT
CTTGAAAGATGAAGAAACACTGACACTATTTCTCATTAAAAACATCGGCCTGTCTGACTC
AGTGGTCTACCTTCTGATCAACTCTCAAGTCCGTCCAGAGCAGTTCGCTCATGGAGTCC
CGGACCTGGCGCTGAAGGACATCGCCTGCAGCGAGGCCCTCCTGGAGCGCTTCATCA
TCTTCAGCCAGAGACGCGGGGCAAAGACGGTGCGCTATGCCCTGTGCTCCCTCTCCCA
GGGCACCCTACAGTGGATAGAAGACACTCTGTATGCCAACGTGGACTTCTTCAAGCTCT
TCCGTGTGCTTCCCACACTCCTAGACAGCCGTTCTCAAGGTATCAATCTGAGATCTTGG
GGAGGAATATTATCTGATATGTCACCAAGAATTCAAGAGTTTATCCATCGGCCGAGTAT
GCAGGACTTGCTGTGGGTGACCAGGCCCCTCATGCAGAATGGTGGTCCAGAGACCTTT
ACAAAGCTGATGGGCATCCTGTCTGACCTCCTGTGTGGCTACCCCGAGGGAGGTGGCT
CTCGGGTGCTCTCCTTCAACTGGTATGAAGACAATAACTATAAGGCCTTTCTGGGGATT
GACTCCACAAGGAAGGATCCTATCTATTCTTATGACAGAAGAACAACATCCTTTTGTAAT
GCATTGATCCAGAGCCTGGAGTCAAATCCTTTAACCAAAATCGCTTGGAGGGCGGCAA
AGCCTTTGCTGATGGGAAAAATCCTGTACACTCCTGATTCACCTGCAGCACGAAGGATA
CTGAAGAATGCCAACTCAACTTTTGAAGAACTGGAACACGTTAGGAAGTTGGTCAAAGC
CTGGGAAGAAGTAGGGCCCCAGATCTGGTACTTCTTTGACAACAGCACACAGATGAAC
ATGATCAGAGATACCCTGGGGAACCCAACAGTAAAAGACTTTTTGAATAGGCAGCTTGG
TGAAGAAGGTATTACTGCTGAAGCCATCCTAAACTTCCTCTACAAGGGCCCTCGGGAAA
GCCAGGCTGACGACATGGCCAACTTCGACTGGAGGGACATATTTAACATCACTGATCG
CACCCTCCGCCTGGTCAATCAATACCTGGAGTGCTTGGTCCTGGATAAGTTTGAAAGCT
ACAATGATGAAACTCAGCTCACCCAACGTGCCCTCTCTCTACTGGAGGAAAACATGTTC
TGGGCCGGAGTGGTATTCCCTGACATGTATCCCTGGACCAGCTCTCTACCACCCCACG
TGAAGTATAAGATCCGAATGGACATAGACGTGGTGGAGAAAACCAATAAGATTAAAGAC
AGGTATTGGGATTCTGGTCCCAGAGCTGATCCCGTGGAAGATTTCCGGTACATCTGGG
GCGGGTTTGCCTATCTGCAGGACATGGTTGAACAGGGGATCACAAGGAGCCAGGTGC
AGGCGGAGGCTCCAGTTGGAATCTACCTCCAGCAGATGCCCTACCCCTGCTTCGTGGA
CGATTCTTTCATGATCATCCTGAACCGCTGTTTCCCTATCTTCATGGTGCTGGCATGGAT
CTACTCTGTCTCCATGACTGTGAAGAGCATCGTCTTGGAGAAGGAGTTGCGACTGAAG
GAGACCTTGAAAAATCAGGGTGTCTCCAATGCAGTGATTTGGTGTACCTGGTTCCTGGA
CAGCTTCTCCATCATGTCGATGAGCATCTTCCTCCTGACGATATTCATCATGCATGGAA
GAATCCTACATTACAGCGACCCATTCATCCTCTTCCTGTTCTTGTTGGCTTTCTCCACTG
CCACCATCATGCTGTGCTTTCTGCTCAGCACCTTCTTCTCCAAGGCCAGTCTGGCAGCA
GCCTGTAGTGGTGTCATCTATTTCACCCTCTACCTGCCACACATCCTGTGCTTCGCCTG

GCAGGACCGCATGACCGCTGAGCTGAAGAAGGCTGTGAGCTTACTGTCTCCGGTGGC
ATTTGGATTTGGCACTGAGTACCTGGTTCGCTTTGAAGAGCAAGGCCTGGGGCTGCAG
TGGAGCAACATCGGGAACAGTCCCACGGAAGGGGACGAATTCAGCTTCCTGCTGTCCA
TGCAGATGATGCTCCTTGATGCTGCTGTCTATGGCTTACTCGCTTGGTACCTTGATCAG
GTGTTTCCAGGAGACTATGGAACCCCACTTCCTTGGTACTTTCTTCTACAAGAGTCGTA
TTGGCTTGGCGGTGAAGGGTGTTCAACCAGAGAAGAAAGAGCCCTGGAAAAGACCGA
GCCCCTAACAGAGGAAACGGAGGATCCAGAGCACCCAGAAGGAATACACGACTCCTTC
TTTGAACGTGAGCATCCAGGGTGGGTTCCTGGGGTATGCGTGAAGAATCTGGTAAAGA
TTTTTGAGCCCTGTGGCCGGCCAGCTGTGGACCGTCTGAACATCACCTTCTACGAGAA
CCAGATCACCGCATTCCTGGGCCACAATGGAGCTGGGAAAACCACCACCTTGTCCATC
CTGACGGGTCTGTTGCCACCAACCTCTGGGACTGTGCTCGTTGGGGGAAGGGACATTG
AAACCAGCCTGGATGCAGTCCGGCAGAGCCTTGGCATGTGTCCACAGCACAACATCCT
GTTCCACCACCTCACGGTGGCTGAGCACATGCTGTTCTATGCCCAGCTGAAAGGAAAG
TCCCAGGAGGAGGCCCAGCTGGAGATGGAAGCCATGTTGGAGGACACAGGCCTCCAC
CACAAGCGGAATGAAGAGGCTCAGGACCTATCAGGTGGCATGCAGAGAAAGCTGTCG
GTTGCCATTGCCTTTGTGGGAGATGCCAAGGTGGTGATTCTGGACGAACCCACCTCTG
GGGTGGACCCTTACTCGAGACGCTCAATCTGGGATCTGCTCCTGAAGTATCGCTCAGG
CAGAACCATCATCATGTCCACTCACCACATGGACGAGGCCGACCTCCTTGGGGACCGC
ATTGCCATCATTGCCCAGGGAAGGCTCTACTGCTCAGGCACCCCACTCTTCCTGAAGA
ACTGCTTTGGCACAGGCTTGTACTTAACCTTGGTGCGCAAGATGAAAAACATCCAGAGC
CAAAGGAAAGGCAGTGAGGGGACCTGCAGCTGCTCGTCTAAGGGTTTCTCCACCACGT
GTCCAGCCCACGTCGATGACCTAACTCCAGAACAAGTCCTGGATGGGGATGTAAATGA
GCTGATGGATGTAGTTCTCCACCATGTTCCAGAGGCAAAGCTGGTGGAGTGCATTGGT
CAAGAACTTATCTTCCTTCTTCCAAATAAGAACTTCAAGCACAGAGCATATGCCAGCCTT
TTCAGAGAGCTGGAGGAGACGCTGGCTGACCTTGGTCTCAGCAGTTTTGGAATTTCTG
ACACTCCCCTGGAAGAGATTTTTCTGAAGGTCACGGAGGATTCTGATTCAGGACCTCTG
TTTGCGGGTGGCGCTCAGCAGAAAAGAGAAAACGTCAACCCCCGACACCCCTGCTTGG
GTCCCAGAGAGAAGGCTGGACAGACACCCCAGGACTCCAATGTCTGCTCCCCAGGGG
CGCCGGCTGCTCACCCAGAGGGCCAGCCTCCCCCAGAGCCAGAGTGCCCAGGCCCG
CAGCTCAACACGGGGACACAGCTGGTCCTCCAGCATGTGCAGGCGCTGCTGGTCAAG
AGATTCCAACACACCATCCGCAGCCACAAGGACTTCCTGGCGCAGATCGTGCTCCCGG
CTACCTTTGTGTTTTTGGCTCTGATGCTTTCTATTGTTATCCCTCCTTTTGGCGAATACC
CCGCTTTGACCCTTCACCCCTGGATATATGGGCAGCAGTACACCTTCTTCAGCATGGAT
GAACCAGGCAGTGAGCAGTTCACGGTACTTGCAGACGTCCTCCTGAATAAGCCAGGCT
TTGGCAACCGCTGCCTGAAGGAAGGGTGGCTTCCGGAGTACCCCTGTGGCAACTCAAC
ACCCTGGAAGACTCCTTCTGTGTCCCCAAACATCACCCAGCTGTTCCAGAAGCAGAAAT
GGACACAGGTCAACCCTTCACCATCCTGCAGGTGCAGCACCAGGGAGAAGCTCACCAT
GCTGCCAGAGTGCCCCGAGGGTGCCGGGGGCCTCCCGCCCCCCCAGAGAACACAGC
GCAGCACGGAAATTCTACAAGACCTGACGGACAGGAACATCTCCGACTTCTTGGTAAAA
ACGTATCCTGCTCTTATAAGAAGCAGCTTAAAGAGCAAATTCTGGGTCAATGAACAGAG
GTATGGAGGAATTTCCATTGGAGGAAAGCTCCCAGTCGTCCCCATCACGGGGGAAGCA
CTTGTTGGGTTTTTAAGCGACCTTGGCCGGATCATGAATGTGAGCGGGGGCCCTATCA
CTAGAGAGGCCTCTAAAGAAATACCTGATTTCCTTAAACATCTAGAAACTGAAGACAACA
TTAAGGTGTGGTTTAATAACAAAGGCTGGCATGCCCTGGTCAGCTTTCTCAATGTGGCC
CACAACGCCATCTTACGGGCCAGCCTGCCTAAGGACAGGAGCCCCGAGGAGTATGGA
ATCACCGTCATTAGCCAACCCCTGAACCTGACCAAGGAGCAGCTCTCAGAGATTACAGT
GCTGACCACTTCAGTGGATGCTGTGGTTGCCATCTGCGTGATTTTCTCCATGTCCTTCG
TCCCAGCCAGCTTTGTCCTTTATTTGATCCAGGAGCGGGTGAACAAATCCAAGCACCTC

CAGTTTATCAGTGGAGTGAGCCCCACCACCTACTGGGTGACCAACTTCCTCTGGGACA
TCATGAATTATTCCGTGAGTGCTGGGCTGGTGGTGGGCATCTTCATCGGGTTTCAGAA
GAAAGCCTACACTTCTCCAGAAAACCTTCCTGCCCTTGTGGCACTGCTCCTGCTGTATG
GATGGGCGGTCATTCCCATGATGTACCCAGCATCCTTCCTGTTTGATGTCCCCAGCACA
GCCTATGTGGCTTTATCTTGTGCTAATCTGTTCATCGGCATCAACAGCAGTGCTATTACC
TTCATCTTGGAATTATTTGAGAATAACCGGACGCTGCTCAGGTTCAACGCCGTGCTGAG
GAAGCTGCTCATTGTCTTCCCCCACTTCTGCCTGGGCCGGGGCCTCATTGACCTTGCA
CTGAGCCAGGCTGTGACAGATGTCTATGCCCGGTTTGGTGAGGAGCACTCTGCAAATC
CGTTCCACTGGGACCTGATTGGGAAGAACCTGTTTGCCATGGTGGTGGAAGGGGTGGT
GTACTTCCTCCTGACCCTGCTGGTCCAGCGCCACTTCTTCCTCTCCCAATGGATTGCCG
AGCCCACTAAGGAGCCCATTGTTGATGAAGATGATGATGTGGCTGAAGAAAGACAAAG
AATTATTACTGGTGGAAATAAAACTGACATCTTAAGGCTACATGAACTAACCAAGATTTA
TCCAGGCACCTCCAGCCCAGCAGTGGACAGGCTGTGTGTCGGAGTTCGCCCTGGAGA
GTGCTTTGGCCTCCTGGGAGTGAATGGTGCCGGCAAAACAACCACATTCAAGATGCTC
ACTGGGGACACCACAGTGACCTCAGGGGATGCCACCGTAGCAGGCAAGAGTATTTTAA
CCAATATTTCTGAAGTCCATCAAAATATGGGCTACTGTCCTCAGTTTGATGCAATTGATG
AGCTGCTCACAGGACGAGAACATCTTTACCTTTATGCCCGGCTTCGAGGTGTACCAGC
AGAAGAAATCGAAAAGGTTGCAAACTGGAGTATTAAGAGCCTGGGCCTGACTGTCTAC
GCCGACTGCCTGGCTGGCACGTACAGTGGGGGCAACAAGCGGAAACTCTCCACAGCC
ATCGCACTCATTGGCTGCCCACCGCTGGTGCTGCTGGATGAGCCCACCACAGGGATG
GACCCCCAGGCACGCCGCATGCTGTGGAACGTCATCGTGAGCATCATCAGAGAAGGG
AGGGCTGTGGTCCTCACATCCCACAGCATGGAAGAATGTGAGGCACTGTGTACCCGGC
TGGCCATCATGGTAAAGGGCGCCTTTCGATGTATGGGCACCATTCAGCATCTCAAGTC
CAAATTTGGAGATGGCTATATCGTCACAATGAAGATCAAATCCCCGAAGGACGACCTGC
TTCCTGACCTGAACCCTGTGGAGCAGTTCTTCCAGGGGAACTTCCCAGGCAGTGTGCA
GAGGGAGAGGCACTACAACATGCTCCAGTTCCAGGTCTCCTCCTCCTCCCTGGCGAGG
ATCTTCCAGCTCCTCCTCTCCCACAAGGACAGCCTGCTCATCGAGGAGTACTCAGTCAC
ACAGACCACACTGGACCAGGTGTTTGTAAATTTTGCTAAACAGCAGACTGAAAGTCATG
ACCTCCCTCTGCACCCTCGAGCTGCTGGAGCCAGTCGACAAGCCCAGGACGCAGCTC
AGGGGAATTCTGGCTCGAGCGGTGGTGGCGGGAGCGGAGGTGGAGGGTCGTCAGGT
GTGACCGGCTACCGGCTGTTCGAGGAGATTCTGTAATCTAGAGTCGGGGCGGCCGGC
CGCTTCGAGCAGACATGATAAGATACATTGATGAGTTTGGACAAACCACAACTAGAATG
CAGTGAAAAAAATGCTTTATTTGTGAAAT

RP2-LgBiT plasmid

GGCCTAACTGGCCGGTACCTGAGTCTAAATGAGTCTTCGGACCTCGCGGGGGCCGCTT
AAGCGGTGGTTAGGGTTTGTCTGACGCGGGGGGAGGGGGAAGGAACGAAACACTCTC
ATTCGGAGGCGGCTCGGGGTTTGGTCTTGGTGGCCACGGGCACGCAGAAGAGCGCCG
CGATCCTCTTAAGCACCCCCCCGCCCTCCGTGGAGGCGGGGGTTTGGTCGGCGGGTG
GTAACTGGCGGGCCGCTGACTCGGGCGGGTCGCGCGCCCCAGAGTGTGACCTTTTCG
GTCTGCTCGCAGACCCCCGGGCGGCGCCGCCGCGGCGGCGACGGGCTCGCTGGGTC
CTAGGCTCCATGGGGACCGTATACGTGGACAGGCTCTGGAGCATCCGCACGACTGCG
GTGATATTACCGGAGACCTTCTGCGGGACGAGCCGGGTCACGCGGCTGACGCGGAGC
GTCCGTTGGGCGACAAACACCAGGACGGGGCACAGGTACACTATCTTGTCACCCGGA
GGCGCGAGGGACTGCAGGAGCTTCAGGGAGTGGCGCAGCTGCTTCATCCCCGTGGCC
CGTTGCTCGCGTTTGCTGGCGGTGTCCCCGGAAGAAATATATTTGCATGTCTTTAGTTC
TATGATGACACAAACCCCGCCCAGCGTCTTGTCATTGGCGAAGTCGAACACGCAGATG
CAGTCGGGGCGGCGCGGTCCCAGGTCCACTTCGCATATTAAGGTGACGCGTGTGGCC

TCGAACACCGAGCGACCCTGCAGCGACCCGCTTAAAAGCTTGGCAATCCGGTACTGTT
GGTAAAGCCACCAGATCTGCTAGCGATCGCCTAAGTGGGAGCTCAGGGGAATTCTGGC
TCGAGCGGTGGTGGCGGGAGCGGAGGTGGAGGGTCGTCAGGTGCCACCATGGGCTG
CTTCTTCTCCAAGAGACGGAAGGCTGACAAGGAGTCGGTCTTCACACTCGAAGATTTC
GTTGGGGACTGGGAACAGACAGCCGCCTACAACCTGGACCAAGTCCTTGAACAGGGA
GGTGTGTCCAGTTTGCTGCAGAATCTCGCCGTGTCCGTAACTCCGATCCAAAGGATTGT
CCGGAGCGGTGAAAATGCCCTGAAGATCGACATCCATGTCATCATCCCGTATGAAGGT
CTGAGCGCCGACCAAATGGCCCAGATCGAAGAGGTGTTTAAGGTGGTGTACCCTGTGG
ATGATCATCACTTTAAGGTGATCCTGCCCTATGGCACACTGGTAATCGACGGGGTTACG
CCGAACATGCTGAACTATTTCGGACGGCCGTATGAAGGCATCGCCGTGTTCGACGGCA
AAAAGATCACTGTAACAGGGACCCTGTGGAACGGCAACAAAATTATCGACGAGCGCCT
GATCACCCCCGACGGCTCCATGCTGTTCCGAGTAACCATCAACAGCTAATCTAGAGTC
GGGGCGGCCGGCCGCTTCGAGCAGACATGATAAGATACATTGATGAGTTTGGACAAAC
CACAACTAGAATGCAGTGAAAAAAATGCTTTATTTGTGAAATTTGTGATGCTATTGCTTT
ATTTGTAACCATTATAAGCTGCAATAAACAAGTTAACAACAACAATTGCATTCATTTTATG
TTTCAGGTTCAGGGGGAGGTGTGGGAGGTTTTTTAAAGCAAGTAAAACCTCTACAAATG
TGGTAAAATCGATAAGGATCCGTCGACCGATGCCCTTGAGAGCCTTCAACCCAGTCAG
CTCCTTCCGGTGGGCGCGGGGCATGACTATCGTCGCCGCACTTATGACTGTCTTCTTT
ATCATGCAACTCGTAGGACAGGTGCCGGCAGCGCTCTTCCGCTTCCTCGCTCACTGAC
TCGCTGCGCTCGGTCGTTCGGCTGCGGCGAGCGGTATCAGCTCACTCAAAGGCGGTA
ATACGGTTATCCACAGAATCAGGGGATAACGCAGGAAAGAACATGTGAGCAAAAGGCC
AGCAAAAGGCCAGGAACCGTAAAAAGGCCGCGTTGCTGGCGTTTTTCCATAGGCTCCG
CCCCCCTGACGAGCATCACAAAAATCGACGCTCAAGTCAGAGGTGGCGAAACCCGACA
GGACTATAAAGATACCAGGCGTTTCCCCCTGGAAGCTCCCTCGTGCGCTCTCCTGTTC
CGACCCTGCCGCTTACCGGATACCTGTCCGCCTTTCTCCCTTCGGGAAGCGTGGCGCT
TTCTCATAGCTCACGCTGTAGGTATCTCAGTTCGGTGTAGGTCGTTCGCTCCAAGCTGG
GCTGTGTGCACGAACCCCCCGTTCAGCCCGACCGCTGCGCCTTATCCGGTAACTATCG
TCTTGAGTCCAACCCGGTAAGACACGACTTATCGCCACTGGCAGCAGCCACTGGTAAC
AGGATTAGCAGAGCGAGGTATGTAGGCGGTGCTACAGAGTTCTTGAAGTGGTGGCCTA
ACTACGGCTACACTAGAAGAACAGTATTTGGTATCTGCGCTCTGCTGAAGCCAGTTACC
TTCGGAAAAAGAGTTGGTAGCTCTTGATCCGGCAAACAAACCACCGCTGGTAGCGGTG
GTTTTTTTGTTTGCAAGCAGCAGATTACGCGCAGAAAAAAAGGATCTCAAGAAGATCCT
TTGATCTTTTCTACGGGGTCTGACGCTCAGTGGAACGAAAACTCACGTTAAGGGATTTT
GGTCATGAGATTATCAAAAAGGATCTTCACCTAGATCCTTTTAAATTAAAAATGAAGTTTT
AAATCAATCTAAAGTATATATGAGTAAACTTGGTCTGACAGCGGCCGCAAATGCTAAAC
CACTGCAGTGGTTACCAGTGCTTGATCAGTGAGGCACCGATCTCAGCGATCTGCCTATT
TCGTTCGTCCATAGTGGCCTGACTCCCCGTCGTGTAGATCACTACGATTCGTGAGGGC
TTACCATCAGGCCCCAGCGCAGCAATGATGCCGCGAGAGCCGCGTTCACCGGCCCCC
GATTTGTCAGCAATGAACCAGCCAGCAGGGAGGGCCGAGCGAAGAAGTGGTCCTGCT
ACTTTGTCCGCCTCCATCCAGTCTATGAGCTGCTGTCGTGATGCTAGAGTAAGAAGTTC
GCCAGTGAGTAGTTTCCGAAGAGTTGTGGCCATTGCTACTGGCATCGTGGTATCACGC
TCGTCGTTCGGTATGGCTTCGTTCAACTCTGGTTCCCAGCGGTCAAGCCGGGTCACAT
GATCACCCATATTATGAAGAAATGCAGTCAGCTCCTTAGGGCCTCCGATCGTTGTCAGA
AGTAAGTTGGCCGCGGTGTTGTCGCTCATGGTAATGGCAGCACTACACAATTCTCTTAC
CGTCATGCCATCCGTAAGATGCTTTTCCGTGACCGGCGAGTACTCAACCAAGTCGTTTT
GTGAGTAGTGTATACGGCGACCAAGCTGCTCTTGCCCGGCGTCTATACGGGACAACAC
CGCGCCACATAGCAGTACTTTGAAAGTGCTCATCATCGGGAATCGTTCTTCGGGGCGG
AAAGACTCAAGGATCTTGCCGCTATTGAGATCCAGTTCGATATAGCCCACTCTTGCACC

CAGTTGATCTTCAGCATCTTTTACTTTCACCAGCGTTTCGGGGTGTGCAAAAACAGGCA
AGCAAAATGCCGCAAAGAAGGGAATGAGTGCGACACGAAAATGTTGGATGCTCATACT
CGTCCTTTTTCAATATTATTGAAGCATTTATCAGGGTTACTAGTACGTCTCTCAAGGATA
AGTAAGTAATATTAAGGTACGGGAGGTATTGGACAGGCCGCAATAAAATATCTTTATTTT
CATTACATCTGTGTGTTGGTTTTTTGTGTGAATCGATAGTACTAACATACGCTCTCCATC
AAAACAAAACGAAACAAAACAAACTAGCAAAATAGGCTGTCCCCAGTGCAAGTGCAGGT
GCCAGAACATTTCTCT

Figure S8. WT-ABCA4-SmBit and RP2-LgBiT plasmids sequences. WT-ABCA4-SmBiT plasmid sequence is shown with WT-ABCA4 in yellow, linker in cyan and SmBiT in magenta. RP2-LgBiT plasmid sequence is displayed with RP2 in red and LgBiT in green.
